# Anti-apoptotic signaling as a cytoprotective mechanism in mammalian hibernation

**DOI:** 10.7717/peerj.29

**Published:** 2013-02-12

**Authors:** Andrew N. Rouble, Joshua Hefler, Hapsatou Mamady, Kenneth B. Storey, Shannon N. Tessier

**Affiliations:** Institute of Biochemistry & Department of Biology, Carleton University, Ottawa, Ontario, Canada K1S 5B6

**Keywords:** Hibernation, Anti-apoptosis, Inhibitor of apoptosis proteins, Cytoprotection, Bcl proteins

## Abstract

In the context of normal cell turnover, apoptosis is a natural phenomenon involved in making essential life and death decisions. Apoptotic pathways balance signals which promote cell death (pro-apoptotic pathways) or counteract these signals (anti-apoptotic pathways). We proposed that changes in anti-apoptotic proteins would occur during mammalian hibernation to aid cell preservation during prolonged torpor under cellular conditions that are highly injurious to most mammals (e.g. low body temperatures, ischemia). Immunoblotting was used to analyze the expression of proteins associated with pro-survival in six tissues of thirteen-lined ground squirrels, *Ictidomys tridecemlineatus*. The brain showed a concerted response to torpor with significant increases in the levels of all anti-apoptotic targets analyzed (Bcl-2, Bcl-xL, BI-1, Mcl-1, cIAP1/2, xIAP) as well as enhanced phosphorylation of Bcl-2 at S70 and T56. Heart responded similarly with most anti-apoptotic proteins elevated significantly during torpor except for Bcl-xL and xIAP that decreased and Mcl-1 that was unaltered. In liver, BI-1 increased whereas cIAP1/2 decreased. In kidney, there was an increase in BI-1, cIAP and xIAP but decreases in Bcl-xL and p-Bcl-2(T56) content. In brown adipose tissue, protein levels of BI-1, cIAP1/2, and xIAP decreased significantly during torpor (compared with euthermia) whereas Bcl-2, Bcl-xL, Mcl-1 were unaltered; however, Bcl-2 showed enhanced phosphorylation at Thr56 but not at Ser70. In skeletal muscle, only xIAP levels changed significantly during torpor (an increase). The data show that anti-apoptotic pathways have organ-specific responses in hibernators with a prominent potential role in heart and brain where coordinated enhancement of anti-apoptotic proteins occurred in response to torpor.

## Introduction

The cellular pathways which regulate programmed cell death have long been recognized as important biological processes. Several forms of programmed cell death exist (e.g. apoptosis, autophagy, necrosis), but apoptosis is a prominent form in mammalian cells and, in vertebrates, apoptosis proceeds largely through the so-called mitochondrial pathway (Spierings et al., 2005). Apoptotic pathways receive information both extrinsically (e.g. from growth factors, hormones, cytokines, toxins) and intrinsically (e.g. in response to nutrient deprivation, hypoxia, viral infection, calcium concentration, etc.) and influence a cell’s commitment to death (Strasser, Cory & Adams, 2011). Ultimately, dysregulation of the molecular signals responsible for promoting versus inhibiting apoptosis has been linked to various disease or degenerative states including atrophy and cancer (Siu, 2009; Strasser, Cory & Adams, 2011).

**Figure 1:**
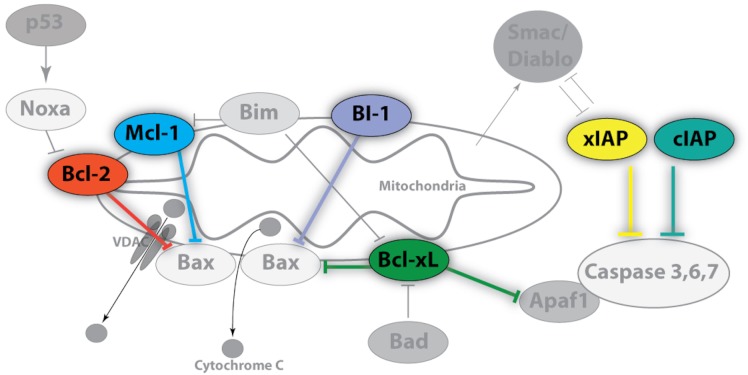
Image of anti-apoptotic proteins at the mitochondrial membrane. Anti-apoptotic proteins, such as Bcl-2, Bcl-xL, Mcl-1, and BI-1, maintain the integrity of the mitochondrial membrane by countering the influences of local pro-apoptotic family members (Bad, Bax, Bim, Apaf1) whereas the inhibitor of apoptosis (IAP) protein family regulates the caspase cascade through a physical interaction which inhibits proteolytic activity. Pro-survival members are highlighted and colour-coded whereas pro-apoptotic members are shown in grayscale. Arrowheads denote positive regulatory effects whereas blunt-ended lines denote negative regulatory effects.

A key initial event in the mitochondrial pathway of apoptosis is mitochondrial outer membrane permeabilization (MOMP) that leads to the release of biomolecules from the organelle including cytochrome c (Green & Kroemer, 2004). As such, the mitochondria lie upstream of irreversible cellular damage and local proteins play roles in either the inhibition or promotion of MOMP (Fig. 1) (Estaquier et al., 2012). A subset of B-cell lymphoma 2 (Bcl-2) family proteins are well-known for their roles in controlling apoptosis at the mitochondria (Chipuk et al., 2010). Bcl-2, Bcl-xL, and Mcl-1 are core pro-survival Bcl-2 family members whereas Bak, Bax, and BAD are pro-apoptotic members (McDonnell et al., 1996). Other local proteins, including BI-1 (Bax inhibitor-1), also regulate cell death via inhibition of pro-apoptotic Bcl-2 members, but are also linked with endoplasmic reticulum (ER) stress (Ishikawa et al., 2011). When pro-apoptotic influences dominate, cytochrome c is released into the cytosol and binds to Apaf-1 (Apoptotic protease activating factor 1) initiating apoptosome formation and activating the caspase cascade (Cain, Bratton & Cohen, 2002). While acting downstream of MOMP and apoptosome formation, selected caspases may also be induced independently of mitochondria-associated events (e.g. TRAIL and TNF death receptors). As a result, additional pro-survival members such as the inhibitor of apoptosis protein (IAP) family, including cIAP1/2 and xIAP, function by interfering with caspase activation as well as by playing a role in NFκB signal transduction (Gyrd-Hansen & Meier, 2010).

The battle between pro- and anti-apoptotic forces is multifaceted and complex, playing a pivotal role in an organism’s response to environmental and cellular insults. Consequently, the use of a wide range of model systems has been an invaluable tool, providing different vantage points, in the field of apoptosis research (Ishizuya-Oka, Hasebe & Shi, 2004; Orme & Meier, 2009; Eimon & Ashkenazi, 2010; Menze et al., 2010; Van Breukelen, Krumschnabel & Podrabsky, 2010; Silva et al., 2011; Wu, Wang & Xue, 2012). This concept is most persuasively demonstrated when important regulatory mechanisms are identified in non-classical model organisms that are found to be dysregulated in human diseases, resulting in putative new intervention strategies for disease treatment. Organisms that face extreme environmental challenges over the course of their life cycle are of particular interest because they endure under conditions that are incompatible with human survival. Stress-tolerant vertebrates include mammalian hibernators, such as ground squirrels, that undergo dramatic physiological and biochemical changes between summer and winter as well as over cycles of torpor and arousal during the winter months (Margesin, Neuner & Storey, 2007; Storey & Storey, 2010).

Mammalian hibernation is characterized by periods of deep torpor where metabolic rate may fall to just 2%–4% of normal and body temperature can be as low as 0–5 °C, resulting in energy savings of up to 88% as compared to the energy that would otherwise be needed to remain euthermic over the winter months (Wang & Lee, 1996). Heart rate, breathing and all other body functions also fall to <5% of normal and, yet, every 1–2 weeks the animals spontaneously arouse back to euthermia (Storey, 2010). Hibernation is not a passive process and important regulatory signals actively promote the hypometabolic state by suppressing ATP-costly cellular activities (e.g. transcription, translation, ion motive ATPases) balanced with comparable suppression of ATP-generating pathways (MacDonald & Storey, 1999; Storey & Storey, 2004; Armstrong & Staples, 2010). In addition, cell preservation strategies (e.g. antioxidant defenses, the unfolded protein response [UPR], heat shock proteins) are enhanced to help preserve macromolecules during prolonged torpor (Mamady & Storey, 2006; Mamady & Storey, 2008; Orr et al., 2009; Storey & Storey, 2010). In essence, hibernators can endure a range of cellular stresses that are known to activate pro-apoptotic proteins in nonhibernating mammals (e.g. nutrient deprivation, ischemia/reperfusion, oxidative damage, low body temperature) and, therefore, they present an opportunity to learn how nature promotes mammalian cell survival under unfavorable conditions.

The present study aimed to identify the responses by anti-apoptotic proteins to torpor in hibernating thirteen-lined ground squirrels, *Ictidomys tridecemlineatus,* in order to uncover the mechanisms employed to resist damage under physiological conditions that most mammals cannot survive. Previous data suggest that hibernators regulate signals that control apoptosis (Chen et al., 2008; Yan et al., 2008; Jani et al., 2011). For example, studies of the brain of the greater horseshoe bat demonstrated that multiple genes that were over-expressed during hibernation were involved in the regulation of cell cycle and apoptosis (Chen et al., 2008). Studies of differential gene expression in arctic ground squirrels found that expression profiles of apoptosis-related genes increased significantly in brown adipose tissue, liver, heart, hypothalamus and skeletal muscle during arousal phases of the torpor-arousal cycle (Yan et al., 2008). These data led us to propose that changes in the expression of anti-apoptotic proteins may play a protective role against the stresses associated with the winter season and that this stress response could be executed via two central mechanisms. Firstly, inhibition of MOMP could occur through the enhanced expression of local mitochondrial proteins such as Bcl-2, Bcl-xL, Mcl-1, and BI-1 (and/or posttranslational modifications of their actions) and, secondly, the direct inhibition of caspase activity could result from increased levels of members of the IAP protein family (e.g. cIAP and xIAP). The present data suggest that anti-apoptotic mechanisms show organ-specific responses which may be attributed to the unique challenges faced by different organs of ground squirrels over torpor-arousal cycles.

## Materials and Methods

### Animals

Hibernation experiments were conducted by the laboratory of Dr. J.M. Hallenbeck at the Animal Hibernation Facility, National Institute of Neurological Disorders and Stroke (NIH, Bethesda, MD), as described by McMullen & Hallenbeck (2010). Briefly, thirteen-lined ground squirrels (*Ictidomys tridecemlineatus)*, weighing 150–300 g, were wild-captured by a United States Department of Agriculture-licensed trapper (TLS Research, Bloomingdale, IL) and transported to the NIH. Animal housing and experimental procedures followed the guidelines set by the NINDS animal care and use committee (ACUC). Animals were individually housed in shoebox cages at 21 °C and fitted with a sensor chip (IPTT-300; Bio Medic Data Systems) injected subcutaneously while anesthetized with 5% isofluorane. Animals were fed standard rodent diet and water *ad libitum* until they gained sufficient lipid stores to enter hibernation. To enable a natural transition into torpor, animals were transferred to an environmental chamber at ∼5 °C in constant darkness. Body temperature (*T*_*b*_), time and respiration rate were monitored and used to determine sampling points. All animals had been through a series of torpor-arousal bouts prior to sampling. Animals were sampled as in McMullen & Hallenbeck (2010) and tissue samples were shipped to Carleton University on dry ice. Tissues were stored at −80 °C until use. EC designates euthermic, cold room; these euthermic squirrels had a stable *T*_*b*_ (∼37 °C) in the 5 °C cold room, were capable of entering torpor but had not done so in the past 72 h. LT designates late torpor; animals that were constantly in deep torpor for at least 5 days with *T*_*b*_ values of 5–8 °C.

### Total protein extraction and preparation

Samples of frozen tissues (brown adipose, skeletal and cardiac muscle, liver, kidney, brain) from two sampling points (EC and LT) were separately extracted (*N* = 4–5 samples from different animals). Tissues were quickly weighed, crushed into small pieces under liquid nitrogen, and then homogenized 1:3 w:v using a Polytron PT10 in ice-cold homogenizing buffer (20 mM Hepes, 200 mM NaCl, 0.1 mM EDTA, 10 mM NaF, 1 mM Na_3_V O_4_, 10 mM β-glycerophosphate) with 1 mM phenylmethylsulfonyl fluoride (BioShop) and 1 µl protease inhibitor cocktail (BioShop) added immediately before homogenization. Each sample was centrifuged for 10 min (12,000 g, 4 °C), the supernatant containing soluble proteins was removed, and the protein concentration was determined by the Coomassie blue dye-binding method (BioRad Laboratories, Hercules, CA) using a MR5000 microplate reader. Samples were adjusted to a constant 4 µg/µl by addition of small amounts of homogenizing buffer and then aliquots were combined 1:1 v:v with 2X SDS loading buffer (100 mM Tris-base pH 6.8, 4% w:v SDS, 20% v:v glycerol, 0.2% w:v bromophenol blue, 10% v:v 2-mercaptoethanol) and boiled. Final protein samples (2 µg/µl) were stored at −40 °C until use.

### Western blotting

Equal amounts of protein from each sample (20–30 µg protein/well) were loaded onto SDS-polyacrylamide gels (SDS-PAGE) or Tris-tricine gels and separated on a BioRad Mini Protean III apparatus. Discontinuous SDS-polyacrylamide gels were used with a 5% stacking gel pH 6.8 and a 10% resolving gel pH 8.8. For Tris-tricine gels, the stacking gel was composed of 375 µl 3.0 M Tris-HCl/SDS (pH 8.45), 253 µl 30% acrylamide, 875 µl water, 15 µl 10% APS, 1.5 µl TEMED whereas the 15% resolving gel contained 1.875 mL 3.0 M Tris-HCl/SDS (pH 8.45), 2.875 mL 30% acrylamide, 316 µl water, 112 µl 10% APS, 3.5 µl TEMED. The Tris-tricine 10X anode buffer was 2 M Tris-HCl, pH 8.8 and the 10X running buffer was 1 M Tris-HCl, 1 M Tricine, 1% w/v SDS, pH 8.3. For detection of Bcl-2, samples were loaded on 15% Tris-tricine gels and run at 30 V for 1 h followed by ∼2 h at 150 V. Other proteins were separated by standard SDS-PAGE using either 12% (Bcl-xL, BI-1) or 10% (cIAP, xIAP, Mcl-1) gels run at 180 V for 45–60 min. Proteins were then transferred to PVDF membrane (0.2 micron PVDF for transfer of Tris-tricine gels and 0.45 µm PVDF for all others) by electroblotting at 160 mA for 1–1.5 h using a transfer buffer containing 25 mM Tris (pH 8.5), 192 mM glycine and 10% v:v methanol at room temperature.

**Figure 2:**
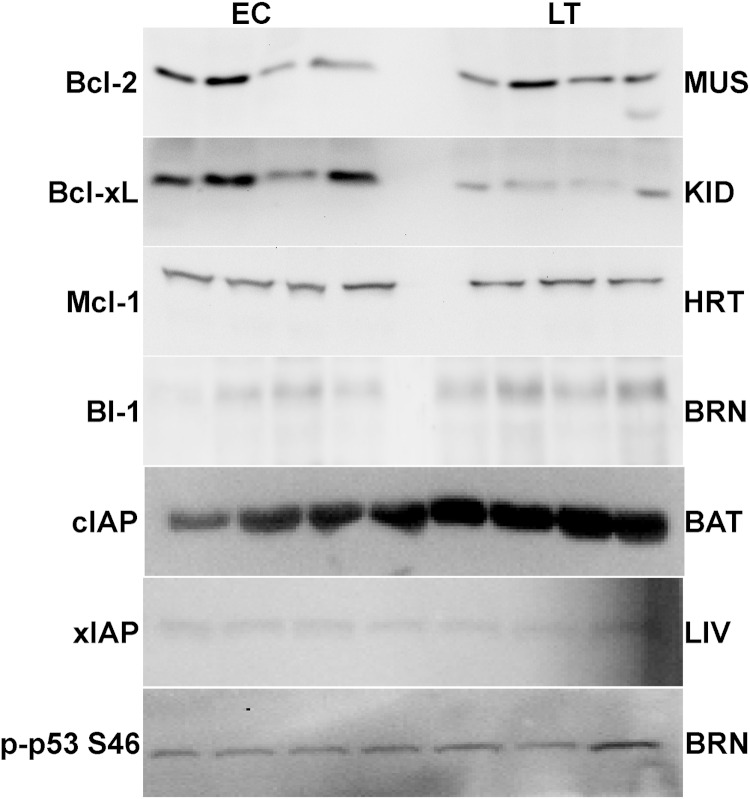
Representative Western blots for extracts from each tissue are shown. The targets of interest are labeled to the left and the tissue to the right of the gel. The antibodies cross-reacted with single strong bands on the immunoblots at the expected molecular masses for the following proteins: Bcl-2 (26 kDa), Bcl-xL (30 kDa), cIAP (69 kDa), xIAP (53 kDa), p53 (53 kDa), Mcl-1 (40 kDa), and BI-1 (27 kDa). In samples from the liver and brain the first 3 bands represent EC state and the next 4 bands represent LT state.

Membranes were blocked with either milk (2%–5% w:v) or polyvinyl alcohol (PVA) made up in TBST (20 mM Tris base, pH 7.6, 140 mM NaCl, 0.05% v:v Tween-20). Those blocked with milk were incubated on a rocker for 20–40 min (Bcl-xL, cIAP, xIAP, p53, Bcl-2 all forms). When PVA was used, incubation was with 1 mg/ml PVA (70–100 kDa or 30–70 kDa) in TBST for 45 s (Mcl-1, BI-1). Membranes were then probed with specific primary antibodies at 4 °C for 12–24 h. Antibodies were carefully selected from suppliers with the following criteria: antibodies cross-react with several model organisms and the epitope correlates with highly conserved protein regions. Antibodies were diluted 1:1000 v:v in TBST (0.05% Tween-20) except for xIAP and cIAP1/2 which were 1:200 v:v dilution. Membranes that had been probed with Bcl-2, p-Bcl-2 S70, p-Bcl-2 T56, Bcl-xL, Mcl-1 or p-p53 S46 antibodies were then incubated with HRP-linked anti-rabbit IgG secondary antibody (1:4000 v:v dilution); HRP-linked anti-goat IgG secondary antibody (1:8000 v:v dilution) was used for cIAP1/2 and BI-1 and HRP-linked anti-mouse IgG secondary antibody (1:2000 v:v dilution) was used for xIAP. All membranes were washed between incubation periods in TBST for ∼10 min per wash. Bands were visualized by enhanced chemiluminescence (H_2_O_2_ and luminol) and then blots were restained using Coomassie blue (0.25% w:v Coomassie brilliant blue, 7.5% v:v acetic acid, 50% methanol). Antibodies specific for mammalian BI-1 and cIAP1/2 were purchased from Santa Cruz, xIAP was from Stressgen, and all others were from Cell Signaling including phospho-specific antibodies detecting Bcl-2 phosphorylated on S70 or T65 and p53 phosphorylated on S46. Antibodies each cross-reacted with single strong bands on the immunoblots at the expected molecular masses for Bcl-2 (26 kDa), Bcl-xL (30 kDa), cIAP (69 kDa), xIAP (53 kDa), p53 (53 kDa), Mcl-1 (40 kDa), and BI-1 (27 kDa) (Fig. 2). Using online bioinformatics tools, it was confirmed that the predicted molecular weight of the target ground squirrel proteins are similar to those from other model organisms.

### Quantification and statistics

Band densities on chemiluminescent immunoblots were visualized using a Chemi-Genius BioImaging system (Syngene, Frederick, MD) and quantified using the associated Gene Tools software. Immunoblot band density in each lane was standardized against the summed intensity of a group of Coomassie stained protein bands in the same lane; these were chosen because they did not show variation between different experimental states and were not located close to the protein bands of interest. Data are expressed as means ± SEM, *n* = 3–4 independent samples from different animals. Statistical testing of standardized band intensities used the Student’s *t*-test (*p* < 0.05).

## Results

### Mitochondria-associated anti-apoptotic proteins in brown adipose tissue

**Figure 3:**
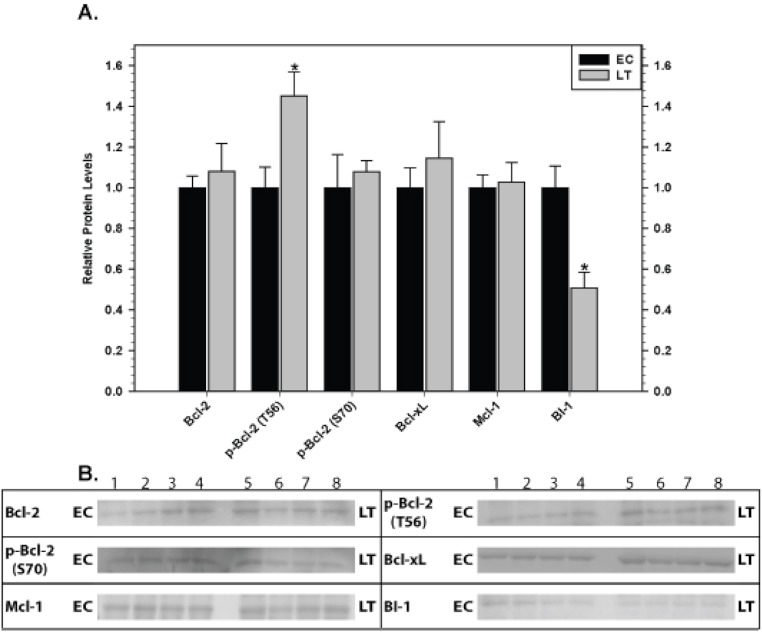
Levels of mitochondria-associated pro-survival proteins in brown adipose tissue of euthermic (EC) and torpid (LT) thirteen-lined ground squirrels. (A) Histogram showing mean relative expression levels of Bcl-2, p-Bcl-2 T56, p-Bcl-2 S70, Bcl-xL, Mcl-1, and BI-1 (± S.E.M., *n* = 4 independent protein isolations from different animals). (B) Representative Western blots are shown and sampling points are labeled to the left and right of the gel; sample numbers (lanes) are labeled along the top indicating 4 control samples (i.e. EC lanes 1, 2, 3, 4) and 4 torpid samples (i.e. LT lanes 5, 6, 7, 8). Data were analyzed using the Student’s *t*-test; * values are significantly different from EC, *p* < 0.05.

Relative levels of pro-survival proteins in brown adipose tissue were measured by immunoblotting comparing euthermic control squirrels (EC) with animals in deep torpor (LT) (Fig. 3). As compared with EC controls, levels of the BI-1 pro-survival protein decreased significantly by 50% during deep torpor. However, total Bcl-2, Bcl-xL and Mcl-1 protein levels did not change. Bcl-2 can be regulated by phosphorylation on selected sites; serine 70 is the best-studied but threonine 56 is another known target (Ruvolo, Deng & May, 2001; Deng et al., 2004). Relative phosphorylation at these two sites was also evaluated. Compared with EC, the relative level of Bcl-2 phosphorylation at T56 in brown adipose increased significantly in deep torpor (by 1.5 fold) whereas phospho-S70 content was unaltered.

### Mitochondria-associated anti-apoptotic proteins in skeletal and cardiac muscles

**Figure 4:**
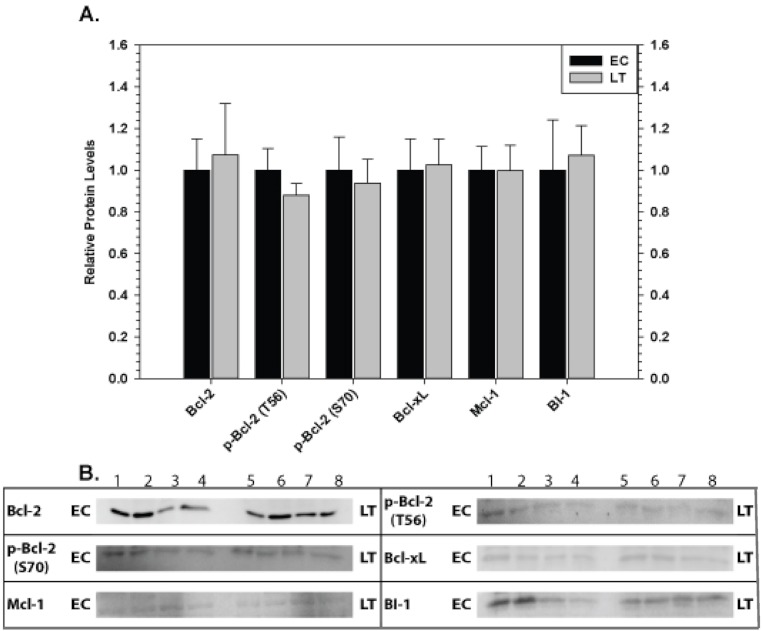
Levels of mitochondria-associated pro-survival proteins in skeletal muscle of euthermic (EC) and torpid (LT) ground squirrels. (A) Histogram showing mean relative expression levels of Bcl-2, p-Bcl-2 T56, p-Bcl-2 S70, Bcl-xL, Mcl-1, and BI-1. (B) Representative Western blots. Other information as in Fig. 3.

**Figure 5:**
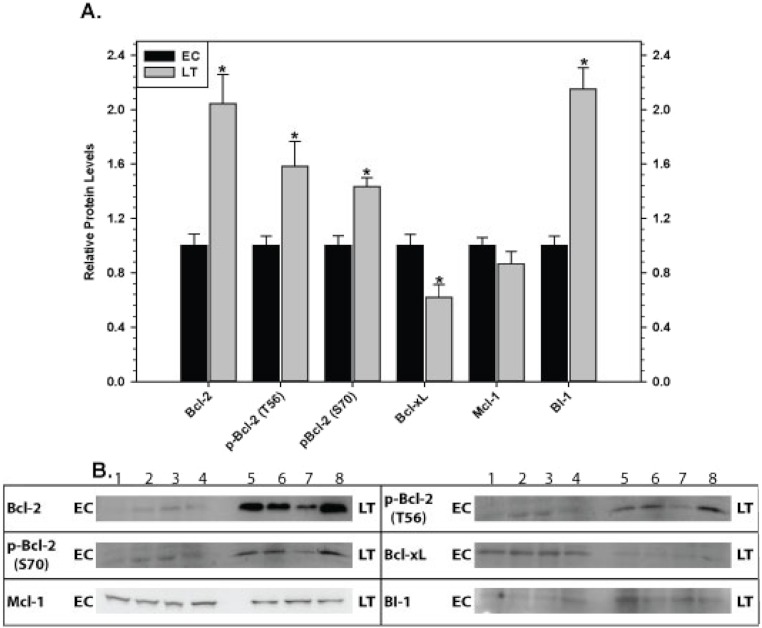
Levels of mitochondria-associated pro-survival proteins in heart of euthermic (EC) and torpid (LT) ground squirrels. (A) Histogram showing mean relative expression levels of Bcl-2, p-Bcl-2 T56, p-Bcl-2 S70, Bcl-xL, Mcl-1, and BI-1. (B) Representative Western blots are shown and sampling points are labeled to the left and right of the gel; sample numbers (lanes) are labeled along the top indicating 4 control samples (i.e. EC lanes 1, 2, 3, 4) and 3–4 torpid samples (i.e. LT lanes 5, 6, 7, 8). Other information as in Fig. 3.

Relative levels of pro-survival proteins were analyzed in ground squirrel skeletal muscle (Fig. 4) and cardiac muscle (Fig. 5) comparing EC and LT states. As compared with controls, none of the pro-survival proteins changed in skeletal muscle during torpor, demonstrating a unified expression pattern in response to torpor. Conversely, cardiac muscle showed the opposite pattern with significant changes recorded during deep torpor for all proteins assessed except Mcl-1. As compared with EC controls, total levels of Bcl-2 and BI-1 increased by 2.0 and 2.2 fold, respectively, during LT whereas Bcl-xL levels decreased to 60% of the control values. Phosphorylation of Bcl-2 was also enhanced during torpor with the relative content of p-Bcl-2 T56 and p-Bcl-2 S70 rising by 1.6 and 1.4 fold, respectively, during LT.

### Mitochondria-associated anti-apoptotic proteins in liver and kidney

**Figure 6:**
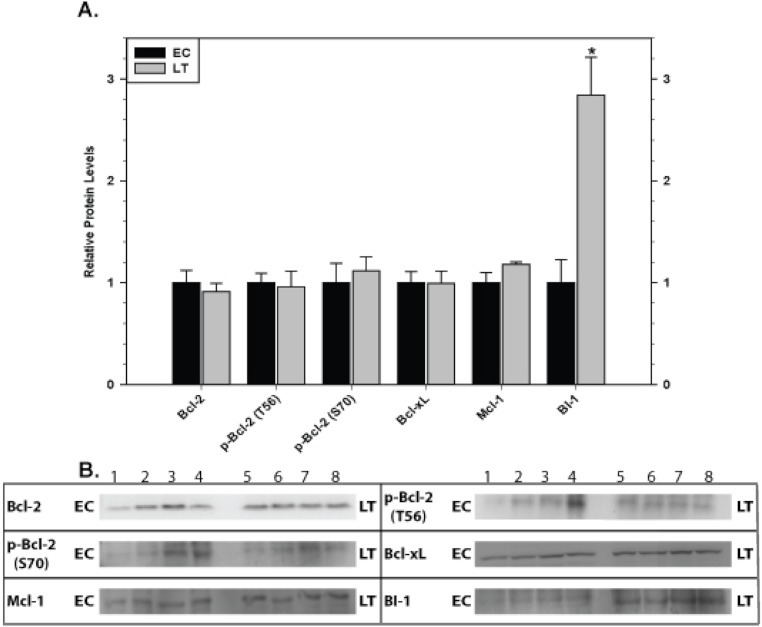
Levels of mitochondria-associated pro-survival proteins in liver of euthermic (EC) and torpid (LT) ground squirrels. (A) Histogram showing mean relative expression levels of Bcl-2, p-Bcl-2 T56, p-Bcl-2 S70, Bcl-xL, Mcl-1, and BI-1. (B) Representative Western blots. Other information as in Fig. 3.

**Figure 7:**
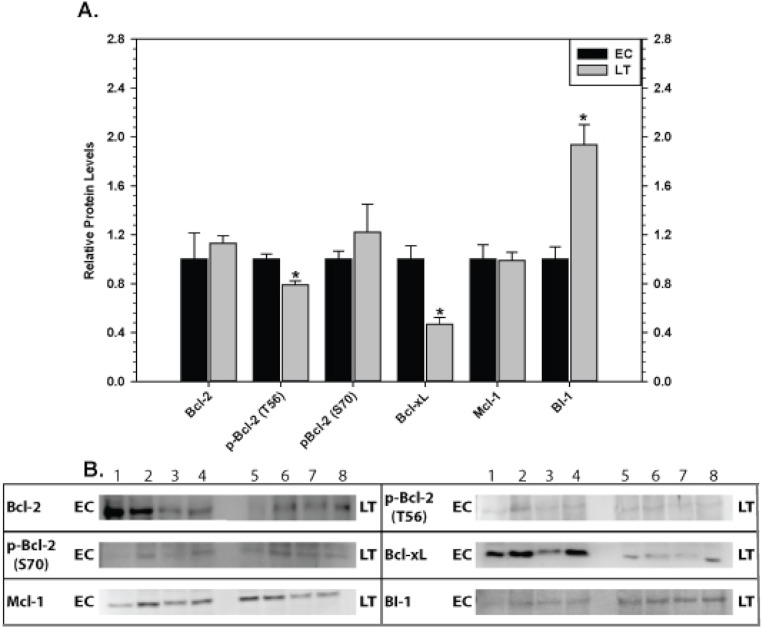
Levels of mitochondria-associated pro-survival proteins in kidney of euthermic (EC) and torpid (LT) ground squirrels. (A) Histogram showing mean relative expression levels of Bcl-2, p-Bcl-2 T56, p-Bcl-2 S70, Bcl-xL, Mcl-1, and BI-1. (B) Representative Western blots. Other information as in Fig. 3.

The effect of torpor on pro-survival proteins in liver and kidney are shown in Figs. 6 and 7, respectively. In liver, the only significant change was a 2.8 fold increase in BI-1 content during LT. In kidney, BI-1 also increased strongly by 1.9 fold during LT whereas Bcl-xL levels decreased to 47%. Phosphorylation of Bcl-2 at T56 also decreased significantly during torpor to 80% of the control value.

### Mitochondria-associated anti-apoptotic proteins in brain

**Figure 8:**
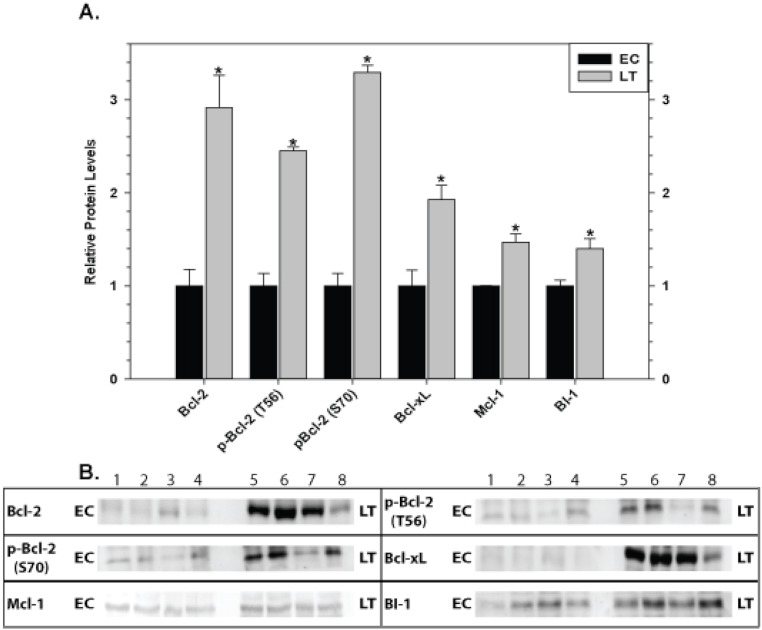
Levels of mitochondria-associated pro-survival proteins in brain of euthermic (EC) and torpid (LT) ground squirrels. (A) Histogram showing mean relative expression levels of Bcl-2, p-Bcl-2 T56, p-Bcl-2 S70, Bcl-xL, Mcl-1, and BI-1. (B) Representative Western blots. Other information as in Fig. 3.

Relative levels of pro-survival proteins in brain are shown in Fig. 8 comparing EC and LT states. Brain showed the strongest and most unified response of all the tissues with significant increases in all pro-survival proteins studied. Bcl-2 was strongly affected with a 2.9 fold increase in total protein as well as comparable increases in p-Bcl-2 S70 (3.3 fold higher) and p-Bcl-2 T56 (2.5 fold higher) contents. Levels of Bcl-xL, Mcl-1, and BI-1 also increased significantly during torpor by 1.9, 1.5, and 1.4 fold, respectively.

### Inhibitor of apoptosis proteins in hibernator tissues

**Figure 9:**
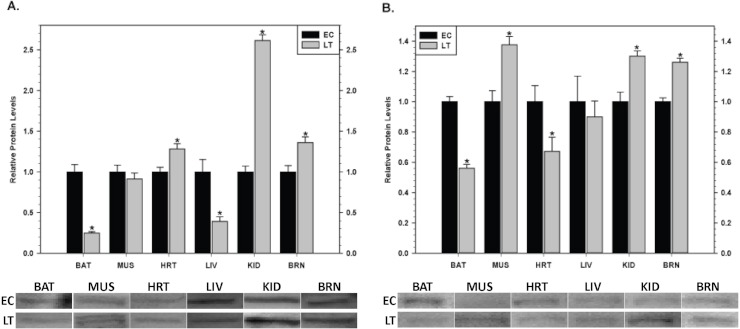
Changes in the protein levels of members of the inhibitor of apoptosis (IAP) protein family comparing euthermic (EC) and hibernating (LT) conditions in ground squirrel tissues. (A) The relative expression levels of cIAP1/2. (B) The relative expression levels of xIAP. Tissues are brown adipose tissue (BAT), skeletal muscle (MUS), heart (HRT), liver (LIV), kidney (KID) and brain (BRN) of thirteen-lined ground squirrels. Representative Western blots are shown with sampling points labeled to the left and tissues labeled above the bands. Histograms show mean standardized band densities (±S.E.M., *n* = 4 independent protein isolations from different animals). Data were analyzed using the Student’s *t*-test; * values are significantly different from EC, *p* < 0.05.

Relative levels of pro-survival IAP proteins involved in the regulation of caspase activity were also quantified during EC and LT (Fig. 9). In brown adipose tissue, levels of cIAP1/2 (Fig. 9A) and xIAP (Fig. 9B) decreased significantly during deep torpor to levels that were just 20 and 60% of EC values, respectively. In skeletal muscle, xIAP levels increased by 1.4 fold during torpor but cIAP1/2 was unchanged. However, in cardiac muscle, cIAP1/2 and xIAP showed opposite expression patterns; cIAP1/2 increased by 1.3 fold and xIAP decreased in LT to 70% as compared with the EC value. In liver, cIAP1/2 expression levels decreased significantly during torpor to just 40% of EC values whereas xIAP was unchanged. A concerted expression profile was observed in kidney with both cIAP1/2 and xIAP increasing significantly in LT by 2.6 and 1.3 fold, respectively. Similarly, both proteins increased significantly in brain during torpor by 1.4 and 1.3 fold, respectively.

### Pro-apoptotic proteins in heart and brain

**Figure 10:**
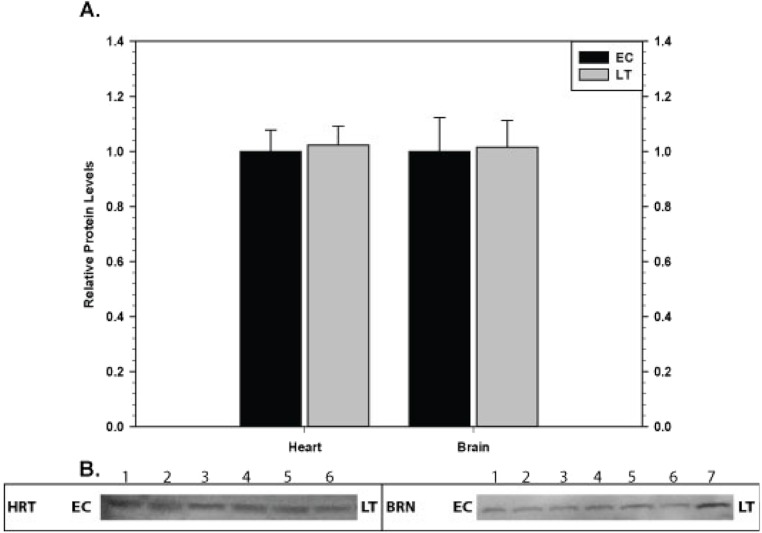
Levels of phospho-p53 (S46) in heart (HRT) and brain (BRN) of thirteen-lined ground squirrels comparing euthermic (EC) and hibernating (LT) conditions. (A) Histogram showing mean relative expression levels of p-p53 (S46) (±S.E.M., *n* = 3–4 independent protein isolations from different animals). (B) Representative Western blots are shown and sampling points are labeled to the left and right of the gel; sample numbers (lanes) are labeled along the top indicating 3 control samples (i.e. EC lanes 1, 2, 3) and 3–4 torpid samples (i.e. LT lanes 4, 5, 6, 7). Data were analyzed using the Student’s *t*-test; * values are significantly different from EC, *p* < 0.05.

Relative levels of the pro-apoptotic protein p53 phosphorylated at S46 were quantified in tissues which showed a strong, concerted anti-apoptosis response comparing EC and LT (Fig. 10). Phosphorylation of p53 at S46 is correlated with cells directed towards apoptosis (Smeenk et al., 2011); therefore, the relative expression levels were used as an indicator of apoptosis in heart and brain. In both tissues, there was no change in p-p53 S46 protein levels comparing EC and LT.

## Discussion

The ground squirrel represents a natural, stress-tolerant model system that may shed light on multiple processes relevant to human health and disease. Cellular mechanisms employed by squirrels that counter, for example, the damaging effects of low body temperatures, ischemia/reperfusion, or muscle disuse could help biomedical researchers to design new preservation techniques to use in tissue/organ storage and transplantation or identify new therapeutic targets for counteracting effects of hypothermia, reperfusion injury, metabolic diseases, atrophy, etc. The present study highlights the responses of pro-survival anti-apoptotic proteins in organs of a mammalian hibernator. Hibernator organs face different challenges over the torpor-arousal cycle. Heart must continue to pump throughout, adjusting to both low body temperature and the increase in force of contraction needed at low heart rates and high blood viscosities during torpor (Dawe & Morrison, 1955; Fahlman, Storey & Storey, 2000; Brauch et al., 2005). Brown adipose tissue and skeletal muscle have limited roles during torpor but are critical to the arousal phase that returns the animal to euthermia, being responsible for nonshivering and shivering thermogenesis, respectively (Wang & Lee, 1996). Other tissues also face unique challenges; the kidneys experience minimal renal flow and reduced urine production (Deavers & Musacchia, 1980), the liver must orchestrate lipolysis, gluconeogenesis from amino acids/glycerol, and urea recycling (Storey & Storey, 2010), and the brain faces strongly reduced cerebral blood flow while remaining crucial for the control of torpor-arousal cycles (Frerichs et al., 1994). The essential roles that these tissues play in the physiological regulation of hibernating mammals suggest the importance of molecular controls that ensure their preservation under stressful conditions. Anti-apoptosis actions may be crucial to long term cell survival in the hypometabolic torpid state, joining other cytoprotective mechanisms (e.g. UPR, heat shock proteins, antioxidant defenses) to help preserve cellular macromolecules and extend viability. This is necessary because the hypometabolic state is characterized by a reduced capacity to replace damaged macromolecules due to the global suppression of transcription and translation. We hypothesized a two-pronged mode of action whereby pro-survival targets involved in the inhibition of MOMP (Blc-2, Bcl-xL, Mcl-1, BI-1) and caspase activity (cIAP1/2, xIAP) would help to sustain tissue viability in the torpid state.

While this study evaluates the response of pro-survival proteins, mounting evidence suggests pro-apoptotic stimuli are suppressed and/or remain constant between control and torpid conditions in the tissues presently studied. In the current study, heart and brain showed no change in the relative expression levels of p-p53 S46 suggesting that apoptotic pathways are not activated. In a stressed state, p53 may be phosphorylated at several key residues resulting in the stabilization and/or activation of transcription factor activity (Smeenk et al., 2011). Although some phosphorylation sites are thought to serve a more general purpose, serine 46 phosphorylation directs the transcription of target genes favoring the pro-apoptotic response (Smeenk et al., 2011). Pro-apoptotic data in other tissues include the following; caspase-3 activity was no different between hibernating and summer kidneys (Jani et al., 2011) and relative expression levels of the pro-apoptotic Bcl-2-associated death promoter (BAD) did not change between hibernating (LT) and control (EC) animals in heart and muscle (H Mamady & KB Storey, unpublished). Studies which evaluate the regulatory mechanisms of pro-apoptotic pathways are still required; however, this data for thirteen-lined ground squirrel in multiple tissues suggests that pro-apoptotic pathways are not activated.

The responses by mitochondria-associated anti-apoptotic proteins varied substantially between the six tissues examined. Total protein levels of Bcl-2, Bcl-xL, Mcl-1 and BI-1 did not change in skeletal muscle of ground squirrels, and three other tissues showed responses by only 1 protein (BI-1 in brown adipose and liver) or two proteins (BI-1, Bcl-xL in kidney). By contrast, changes in mitochondria-associated anti-apoptotic proteins were substantial in heart and brain. Cardiac muscle showed a strong pro-survival response with a significant increase in all mitochondria-associated proteins measured except for Bcl-xL (significant decrease) and Mcl-1 (unaltered) (Fig. 5). Generally, these data suggest that anti-apoptotic mechanisms help preserve the integrity of heart tissue during hibernation, possibly in response to increased stress experienced as a result of cold temperatures and/or elevated cardiac muscle contraction strength during this period. In brain, expression levels of all four proteins increased during LT to levels that were 1.4–2.9 fold higher than in EC. The concerted and strong response by all of these anti-apoptotic proteins in brain may reflect a need for focused neuroprotection during torpor. Furthermore, both heart and brain showed increased phosphorylation of Bcl-2 at both the T56 and S70 sites.

The most prominent response to torpor across ground squirrel tissues was a significant change in BI-1 levels in four of the six tissues examined; levels rose significantly by 1.4–2.8 fold in heart, liver, kidney and brain but decreased by 50% in brown adipose and remained unchanged in muscle. BI-1 protects cells against BAX-induced apoptosis, and given the elevation of BI-1 in four tissues during torpor, this argues that attention to BAX regulation may be one of the most important anti-apoptotic actions needed in hibernator organs. BI-1 is important in regulating Ca^2+^ release from intracellular stores and a negative regulator of the endoplasmic reticulum (ER) stress sensor IRE1α (Henke et al., 2011). Sustained release of Ca^2+^ from ER stores is well-known to trigger apoptosis and the inability to maintain Ca^2+^ homeostasis at low temperatures is a major cause of hypothermic injury in nonhibernating mammals, including man (Hochachka, 1986). A consistent element of metabolic rate depression across phylogeny is the coordinated regulation and suppression of ion channels and ATP-driven membrane ion pumps so that ion homeostasis and membrane potential difference is maintained across both plasma and organelle membranes (Hochachka, 1986; Storey & Storey, 2007). Controlled suppression of both the Na^+^K^+^ ATPase and the sarco(endo)plasmic reticulum Ca^2+^ pump (SERCA) that returns Ca^2+^ into the ER has been well-documented in multiple hypometabolic systems, including hibernators. Indeed, ground squirrels show coordinated regulation of SERCA and other Ca^2+^ -related proteins in the ER (Malysheva et al., 2001). Hence, we can propose that BI-1 may be involved not just in anti-apoptosis action at the mitochondria but also in the regulation of Ca^2+^ fluxes in the ER, potentially contributing to the temperature-dependent regulation of Ca^2+^ fluxes that could otherwise cause damage to hibernator cells as their Tb changes over a wide range from 37 °C down to near 4 °C. Additionally, Chae et al. (2004) discovered that mouse cells deficient in BI-1 displayed a hypersensitivity to apoptosis induced specifically by ER stress. Indeed, studies of selected ground squirrel tissues measured markers of ER stress such as the ATF4 transcription factor (activates genes that participate in the UPR), its nuclear co-activator (the phosphorylated form of CREB-1), and GRP78 (the main ER chaperone), all of which provided evidence of ER-related stress during torpor (Mamady & Storey, 2006; Mamady & Storey, 2008).

Notably, however, BI-1 responded differently in the two thermogenic organs, decreasing in brown adipose and not changing in skeletal muscle. Furthermore, total protein levels of the other mitochondria-associated anti-apoptotic targets that are involved in MOMP were unaltered (Bcl-2, Bcl-xL, Mcl-1) in these two tissues during torpor. This may suggest a different approach to anti-apoptotic regulation in the thermogenic organs such as reliance on different anti-apoptotic regulators or a controlled suppression of pro-apoptotic proteins. In line with the idea of alternative anti-apoptotic mechanisms, selected heat shock proteins (Hsp27, Hsp70, Hsp90) have been implicated in the inhibition of apoptosis via a physical interaction with Apaf-1, resulting in caspase inhibition (Saleh et al., 2000; Xanthoudakis & Nicholson, 2000). Notably, HSP70 levels in skeletal muscle of torpid ground squirrels were 50% higher than in euthermic controls, providing some initial support for the idea that an alternative mode of anti-apoptosis may be present in muscle (K Yan & KB Storey, unpublished data; Storey & Storey, 2011).

Protein phosphorylation is an important regulatory mechanism in the pro-survival function of Bcl-2 (Ruvolo, Deng & May, 2001). The best consensus of available data is that Bcl-2 phosphorylation at serine 70 enhances its anti-apoptotic potential (Horiuchi et al., 1997; Ito et al., 1997; Deng et al., 2000; Deng et al., 2004) but the consequences of threonine-56 phosphorylation are still being debated (Srivastava et al., 1999; Deng et al., 2004; Muscarella & Bloom, 2008). These conflicting responses at different sites might be resolved when it is considered that phosphorylation at multiple sites may be present at any given time and that these modifications must be collectively considered when attempting to isolate their functional significance. Other possible explanations may stem from the intimate relationship between apoptosis and other stress-responsive pathways. For example, Furukawa et al. (2000) discovered that Bcl-2 phosphorylation at T56 by Cdc2 kinase correlated with the accumulation of cells in G(2)/M phase and did not relate to levels of apoptosis. Since cell cycle inhibition commonly occurs in response to cellular stress as well as during multiple forms of hypometabolism, including hibernation (Wu & Storey, 2012), Bcl-2 may actually have a dual function, acting to inhibit both apoptosis and cell proliferation when conditions are unfavorable. This could contribute to both metabolic arrest and extending the half-life of macromolecules and cells in the torpid state.

Increases in the phosphorylation state of Bcl-2 at S70 and T56 occurred in both heart and brain during torpor together with similar increases in total Bcl-2 protein levels. It seems significant that the two organs (brain, heart) with elevated Bcl-2 and phospho-Bcl-2 contents are also the two most oxygen-dependent organs as well as being critically important organs that must remain functional and viable throughout torpor to ensure animal survival. Chen & Pervaiz (2007) proposed that Bcl-2 was involved in fine-tuning the balance between mitochondrial oxygen consumption for energy production and reactive oxygen species (ROS) generation. Cells overexpressing Bcl-2 had higher cytochrome oxidase (COX) activity and respiration and Bcl-2 was also able to regulate these in the face of rising ROS levels. They proposed that Bcl-2 helps to adjust mitochondrial respiration to the demands for energy without incurring harmful increases in ROS. Significantly, previous studies with ground squirrels have documented enhanced expression of the mitochondria-encoded subunits of electron transport chain (ETS) enzymes in heart during torpor including COX 1 (complex IV) (Hittel & Storey, 2002) and subunit 2 of NADH–ubiquinone oxidoreductase (ND2; complex I), complex I being the main site of superoxide production (Fahlman, Storey & Storey, 2000). Thus, we can propose that in addition to anti-apoptosis action, elevated expression of Bcl-2 in hibernator heart (and probably brain as well) may be intimately involved in adjusting the balance of oxygen consumption between complexes I and IV of the ETS that may otherwise be disrupted by the effects of low Tb and/or entry into the hypometabolic state. Indeed, altered expression of mitochondria-encoded subunits of ETS complexes is widespread among hypometabolic systems (Storey & Storey, 2004).

Members of the IAP protein family also showed tissue specific responses to hibernation in ground squirrels. Three tissues showed an increase in xIAP protein levels (muscle, kidney, brain) and/or cIAP1/2 protein levels (heart, kidney, brain), suggesting the possibility that inhibition of caspase activity is a critical regulatory mechanism which enhances survival in these tissues. This regulatory mechanism may be especially important for hibernator skeletal muscle since xIAP was the only anti-apoptotic protein that increased in this tissue, among all of the mitochondria-associated and IAP-related proteins that were measured. In addition, increases in IAP protein levels may be related to inhibiting ER-induced apoptosis through caspase-7 which is activated under ER stress and binds directly to xIAP (Gyrd-Hansen & Meier, 2010). As a result, these data may highlight yet another important point of convergence between apoptosis and ER-stress during hibernation whereby xIAP and BI-1 expression may promote survival as a result of ER-induced stress. Conversely, heart showed a decrease in xIAP expression, liver showed no change in xIAP and a decrease in cIAP1/2, and brown adipose tissue showed a decrease in both IAPs. These data suggest the possibility that maintaining stable levels of xIAP/cIAP is sufficient to inhibit caspase activity, caspase activity is inhibited by other mechanisms or a decrease in IAPs is matched with a decrease in the activity/expression of the downstream effectors.

The current data suggest tissue-specific regulation of anti-apoptotic pathways during hibernation, with a prominent potential need for anti-apoptotic action in cardiac muscle and brain. The activation of pro-survival pathways may function as a mechanism to help preserve hibernator cells from stresses associated with the torpid state. The molecular mechanism of preservation involves both the regulation of mitochondria-associated proteins and the inhibitor of apoptosis protein family. Of particular importance during torpor may be protection of cells from apoptosis as a result of ER-stress, but protection from other inducers of cell death is also likely involved.
